# Electrochemical Reduction of Carbon Dioxide on Graphene-Based Catalysts

**DOI:** 10.3390/molecules26030572

**Published:** 2021-01-22

**Authors:** Stefan Delgado, María del Carmen Arévalo, Elena Pastor, Gonzalo García

**Affiliations:** Instituto de Materiales y Nanotecnología, Departamento de Química, Universidad de La Laguna, P.O. Box 456, 38200 La Laguna, Santa Cruz de Tenerife, Spain; sdelgadb@ull.edu.es (S.D.); carevalo@ull.edu.es (M.d.C.A.)

**Keywords:** CO_2_, graphene, HER, electrocatalysts, catalysis, Cu, nanoparticles

## Abstract

The current environmental situation requires taking actions regarding processes for energy production, thus promoting renewable energies, which must be complemented with the development of routes to reduce pollution, such as the capture and storage of CO_2_. Graphene materials have been chosen for their unique properties to be used either as electrocatalyst or as catalyst support (mainly for non-noble metals) that develop adequate efficiencies for this reaction. This review focuses on comparing experimental and theoretical results of the electrochemical reduction reaction of carbon dioxide (ECO_2_RR) described in the scientific literature to establish a correlation between them. This work aims to establish the state of the art on the electrochemical reduction of carbon dioxide on graphene-based catalysts.

## 1. Introduction

Over the last few decades, the drastic increase of carbon dioxide into the atmosphere has caused global warming and the acidification of the oceans, among other environmental problems, thus leading to climate change. The accelerated consumption of fossil fuels has been the priority factor in these problems, but it has not only caused a crisis in the environmental field but also brought an energy crisis. This is a fact that also affects health because air pollution is the sixth-leading cause of death, causing over two million premature deaths worldwide in addition to increasing asthma, respiratory diseases, diseases cardiovascular disease, cancer, etc. [1]. To put an end to this environmental situation, the Paris Agreement, which aims to keep the global temperature rise below 2 °C by 2100 and limit the temperature increase to 1.5 °C above pre-industrial levels, was drawn up in 2015 [2].

For this, complementary strategies are needed to the transition towards renewable energies, including the capture and storage of CO_2_, which help, together with natural processes (photosynthesis and underground mineralization), to balance the presence of CO_2_ in the atmosphere [3]. Carbon capture and use (CCU) has arisen to reduce carbon emissions by using CO_2_ as a raw material. Some examples include the production of methanol, dimethyl carbonate, and sodium carbonate; however, not all of these are sustainable. In this regard, most of these processes require significant energy inputs that carry carbon footprints themselves. Due to these problems related to economic viability, it is difficult to analyze the sustainability of CCU technology [4]. 

Therefore, it is necessary to develop innovative strategies that are capable of converting carbon dioxide into products of energy value (e.g., hydrocarbons and alcohol), thus increasing its applications while reducing emissions. Among the possible conversion products, formic acid has been proposed as a suitable material for the combination of hydrogen storage and CO_2_ fixation. This compound is easy to store and transport, can be used directly in direct formic acid fuel cells (DFAFCs), and releases H_2_ at room temperature using suitable metal catalysts [5,6,7]. Other oxygenated products, such as methanol and ethanol, are promising due to their high energy density, safety, and easy storage; additionally, like formic acid, they present the possibility of working directly in fuel cells. On the other hand, ethylene has enormous added value [8,9]. Of the different ways of converting CO_2_, chemical, photochemical, and electrochemical reductions stand out. Specifically, the electrochemical reduction process has been extensively investigated in recent years [10].

CO_2_ is a very stable molecule under environmental conditions, and its electrochemical reduction reaction (ECO_2_RR) is an endothermic process that requires the participation of multiple electrons and protons. Furthermore, the reaction intermediates of the different stages form various products in turn, depending on the material used as cathode and the applied potential (reduction potential values for Equations (1)–(10) are given relative to a reversible hydrogen electrode (RHE) at pH 7, 1.0 atm, and 25 °C in aqueous solution) [11,12]. Due to this, the selectivity for the desired products is not usually very high, and it is also conditioned by the hydrogen evolution reaction (HER; E^0^ = 0.000 V), which competes with the reaction under study. This currently raises the following challenges for the ECO_2_RR: the need for an increased reaction efficiency, the need for a decreased overpotential to overcome energy barriers and suppress the HER, and the need to obtain moderately high current densities for commercial applications [13,14].

(1) $${\mathrm{CO}}_{2}\left(g\right)+2H^{+}+2e^{-}\to \mathrm{eHCOOH}\;\left(l\right)\;E^{0}\;=\;-0.02\;V$$

(2) $${\mathrm{CO}}_{2}\left(g\right)+2H^{+}+2e^{-}\to \mathrm{eeq}\;\left(g\right)+H_{2}O\;\left(l\right)\;E^{0}\;=\;-0.10\;V$$

(3) $${\mathrm{CO}}_{2}\left(g\right)+8H^{+}+8e^{-}\to {\mathrm{eCH}}_{4}\left(g\right)+2H_{2}O\;\left(l\right)\;E^{0}\;=\;0.17\;V$$

(4) $$2{\mathrm{CO}}_{2}+12H^{+}+12e^{-}\to 2C_{2}H_{4}\left(g\right)+4H_{2}O\;\left(l\right)\;E^{0}\;=\;0.08\;V$$

(5) $${\mathrm{CO}}_{2}\left(g\right)+6H^{+}+6e^{-}\to {\mathrm{eCH}}_{3}\mathrm{OH}\left(l\right)+H_{2}O\;\left(l\right)\;E^{0}\;=\;0.02\;V$$

(6) $$2{\mathrm{CO}}_{2}\left(g\right)+12H^{+}+12e^{-}\to 2C_{2}H_{5}\mathrm{OH}\left(l\right)+3H_{2}O\;\left(l\right)\;E^{0}\;=\;0.09\;V$$

(7) $${\mathrm{CO}}_{2}\left(g\right)+4H^{+}+4e^{-}\to \mathrm{eeq}\;\mathrm{Eq}\left(l\right)+H_{2}O\;\left(l\right)\;E^{0}\;=\;-0.07\;V$$

(8) $$2{\mathrm{CO}}_{2}\left(g\right)+2H^{+}+2e^{-}\to {\mathrm{eH}}_{2}C_{2}O_{4}\left(l\right)\;E^{0}\;=\;-0.50\;V$$

(9) $$2{\mathrm{CO}}_{2}+14H^{+}+14e^{-}\to 4C_{2}H_{6}\left(g\right)+4H_{2}O\;\left(l\right)\;E^{0}\;=\;0.14\;V$$

(10) $${\mathrm{CO}}_{2}\left(g\right)+e^{-}\to {\;\mathrm{CO}}_{2}{}^{•-}\;E^{0}\;=\;-1.900\;V$$

For these reasons, current research is focused on the development of electrocatalysts that are chemically stable and inexpensive, have an appropriate shelf life, have an adequate product selectivity to promote their formation, and (ultimately) have the ability to operate at industry-acceptable current densities [13,15]. Various catalysts have been developed to improve the energy efficiency of the electrochemical reduction of CO_2_ with metals from the d-block (Cu, Co Pt, Fe, Au, Pd, Ag, Zn, Ni, etc.) and from the p-block (In, Sn, Pb, Bi, etc.) [10,16,17,18,19,20]. Specifically, Cu is a unique metallic catalyst for this reaction because it can efficiently reduce CO_2_ to hydrocarbons and oxygenates such as alcohols (either methanol or ethanol), carbon monoxide, and formic acid/format [8,9,21].

**Figure 1:**
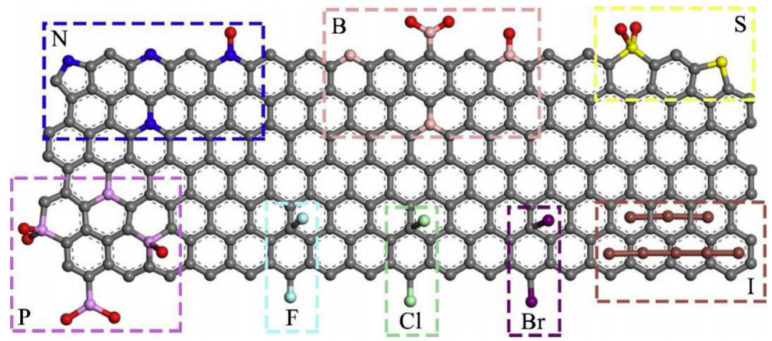
Different possibilities of heteroatom-doped graphene materials. Reproduced with permission [11].

However, in certain cases, excessive overpotentials are required and current densities are not very high. Additionally, for other reasons such as toxicity and the cost of metals, it is intended to develop catalysts without noble metals that can generate the products with acceptable efficiency and conditions [7,22,23,24]. To achieve these catalysts, carbon-based materials are being investigated with special interest due to their wide range of possible nanostructures and because they may adsorb hydrogen and suppress the HER at low overpotentials [25]. Among the wide variety of these materials, graphene (G) and graphene materials (GMs) have become particularly important. In terms of electrochemical activity, pristine graphene is not active for the CO_2_ electroreduction. However, the electrocatalytic activity is enhanced after doping with oxygenated groups and other heteroatoms (Figure 1) [7,26]. Nitrogen was used with the first metal-free G-based catalyst for the ECO_2_RR, and it is the most widely used heteroatom [22,24,25], although there have been studies with others such as B [23], P [15], S [7], F, Cl, Br, and I [11].

### Reaction Mechanism: Key Intermediates, Rate-Determining Steps and Products

The single electron reduction of CO_2_ to a CO_2_^●−^ radical occurs at E^0^ = −1.90 V vs. RHE (Equation (10)) with a high energy contribution so that it accepts an electron, which causes the molecule to lose its initial linear structure and generate a bent radical anion. The lifetime of this radical is very short, thus making it difficult to clearly know its structure. Despite this, it has been established that, for Cu—which is the most studied metal (although it can be generalized for Ag and Au, given their similarity in terms of reaction kinetics and electronic properties)—the bond angle and the distance change, giving rise to the carboxylate intermediate η^2^ (C,O)-CO_2_^•−^ adsorbed on the metal surface [11,27,28,29].

Proton-coupled multi-electron steps for the ECO_2_RR are generally more favorable than single electron reductions due to the more thermodynamically stable molecules that are produced (Equations (1)–(9)). Furthermore, these reactions are influenced by the pH of the electrolyte, and diverse species may therefore be promoted by a specific concentration of protons in the solution [30]. The species produced depend on the catalyst surface nature, e.g., if the CO_2_ adsorption occurs trough carbon, OCHO* is obtained, but if it is trough oxygen, COOH* is formed (* represents an adsorbed species). Hydrogen adsorption and the HER may also occur at the same potential range than the ECO_2_RR, and a competition between both reactions may consequently happen. Then, it is possible to reach a whole series of C_1_ compounds such as HCOOH, HCHO, CH_3_OH, and CH_4_ through both routes. To produce C_2+_ (hydrocarbons and oxygenate species, e.g., C_2_H_4_, C_2_H_6_, and C_2_H_5_OH), the coupling of C_1_ intermediates is required so that a C–C bond is formed [27,28,31].

Another important adsorbed intermediate is *CO, which is the common species for most of the products that are subsequently generated because of its stability. Its protonation can occur trough carbon in order to generate CHO* or trough oxygen to obtain COH*, but the first option is more common. This protonation is one of the most important rate-determining steps [28]. It should be noted that a strong CO* bonding poisons the catalyst, and the HER is preferred. On the other hand, weak CO* adsorption can result in CO desorption, and the reduction will therefore not continue towards other C_1_ products [13].

**Figure 2:**
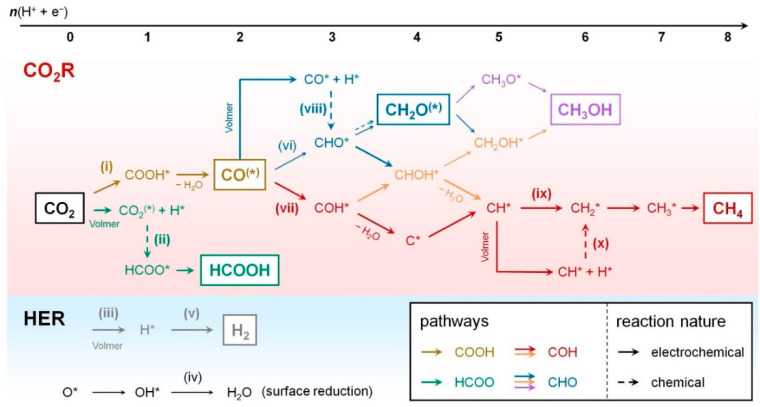
Scheme of the reaction pathways and key intermediates for different CO_2_ electroreduction products. Reproduced with permission [32]. HER: hydrogen evolution reaction.

Figure 2 depicts two pathways to generate *CHO. One of them is through the following species: COOH*, CO*, and CHO*. The other one is through the following species: OCHO*, HCOOH* and CHO*. In this regard, thermodynamics dictate that OCHO* is more favorable than COOH*, although the latter has been more reported in the literature [27].

Another crucial anion that is formed as an intermediate is the dimer (CO)_2_^−^, which has a relevant role in the mechanisms for the formation of C_2_ species. Proton transfer only occurs once the dimer has formed; consequently, dimer formation is the step that limits the rate of the reaction for the formation of these species with two C atoms [30].

## 2. Graphene-Based Catalysts

### 2.1. Metal-Free Graphene-Based Electrocatalysts

Most of the works reported in the literature have studied the ECO_2_RR with graphene materials before preparing the composite with the metal, thus serving as a reference. In general, the ECO_2_RR results of metal-free graphene materials are bad, and the efficiencies are low. Thus, in the work of Huang et al., partially-oxidized, 5-nm cobalt nanoparticles (PO-5 nm Co) dispersed on a single-layer nitrogen-doped graphene (SL-NG) (denoted as PO-5 nm Co/SL-NG) were synthesized and employed for the ECO_2_RR [19]. SL-NG activity is even worse than for PO-Co, and only PO-Co/SL-NG is interesting due to the synergistic effect that occurs after its combination. Something similar was obtained in the work of Ning et al. with cuprous oxide supported on reduced graphene oxide (Cu_2_O/rGO) and cuprous oxide supported on nitrogen-doped reduced graphene oxide (Cu_2_O/N-rGO) [9]. The authors added additional information with data for rGO and N-rGO. The results were not good either because for both GMs, the main product was H_2_. Because of these data, it can be concluded that these GMs are not active materials for the ECO_2_RR, even after being doped with N [9].

**Figure 3:**
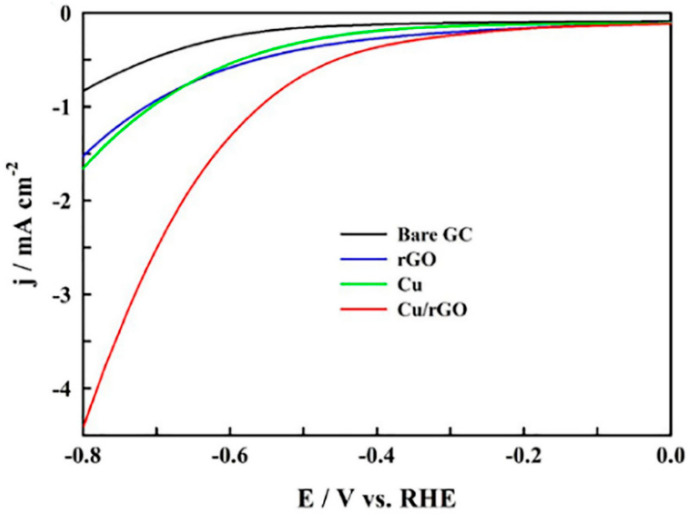
Linear sweep voltammogram (LSV) for glassy carbon (GC), rGO, Cu nanoparticles (NPs), and Cu/rGO in a 0.1 M NaHCO_3_ solution at a scan rate of 20 mVs^−1^. Reproduced with permission [33]. RHE: reversible hydrogen electrode.

Other studies have confirmed this fact [33,34,35]. In the work of Xiong et al. [34], Cu-Sn catalysts were supported on N-doped graphene (NG). For this material, the majority product in the ECO_2_RR is H_2_, and the faradaic efficiency (FE) for this gas reaches 79% at −1.0 V vs. RHE, while it decreases to 68% and 42%, respectively, for Cu/NG and Sn/NG following the previous trend that proves the low activity of GMs for the reaction under study [34]. Additionally, in the work of Hossain et al. [33], in which they deposited Cu nanoparticles (NPs) on rGO, the researchers showed that the catalytic activity of rGO is very similar to that of unsupported Cu NPs, both being very low (Figure 3).

**Figure 4:**
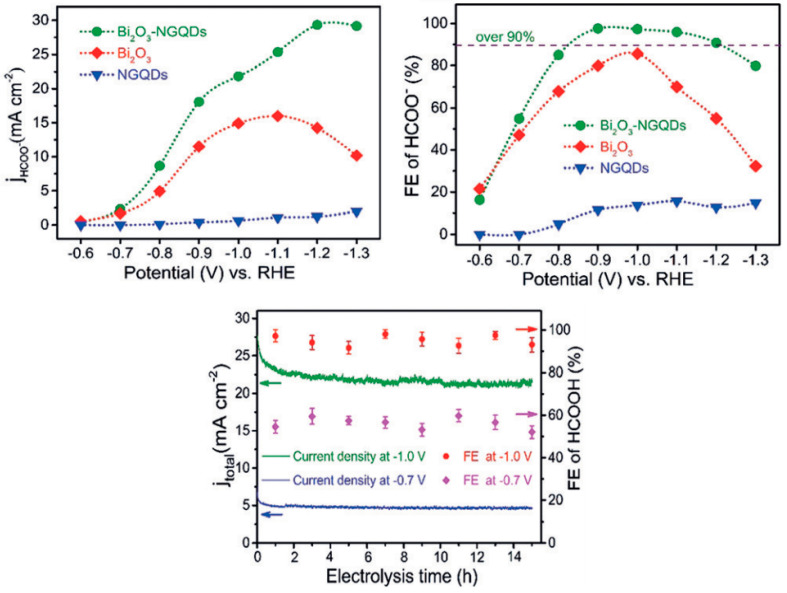
Faradaic efficiency (FE) and formate partial current density for Bi_2_O_3_-NGQD (nitrogen-doped graphene quantum dot), Bi_2_O_3_, and NGQD at different potentials (upper panels) and long-term electrolysis experiments of Bi_2_O_3_-NGQD recorded at −0.7 V and −1.0 V (bottom panel) in a 0.5 M KHCO_3_ electrolyte. Reproduced with permission [35].

Chen et al. developed a 2D/0D composite catalyst formed from bismuth oxide nanosheets and nitrogen-doped graphene quantum dots (Bi_2_O_3_-NGQDs) [35]. The electrochemical response of Bi_2_O_3_ and Bi_2_O_3_-NGQDs is shown in Figure 4 and is compared with that of the graphene material, NGQDs. By comparing the current densities and the FEs for formate production, it was found that the activity of the NGQDs is extremely low, with an FE of around 15%, while the Bi_2_O_3_ and Bi_2_O_3_-NGQD present FEs close to 85% and 100%, respectively, at a potential of −0.9 V (vs. RHE) [35].

Despite the low activities shown in the works mentioned above, there are several strategies to design efficient metal-free electrocatalysts for the ECO_2_RR. For instance, Wu et al. prepared NGQDs that exhibit a high total carbon dioxide reduction efficiency of up to 90%, with selectivity for conversions to ethylene and ethanol reaching 45% in 1 M KOH [36]. Furthermore, the amounts of C_2_ and C_3_ species produced are comparable to those of electrocatalysts based on Cu NPs. The great difference in the results employing similar materials may be related to the synthetic method, the pH, and the counterion, among other factors.

**Figure 5:**
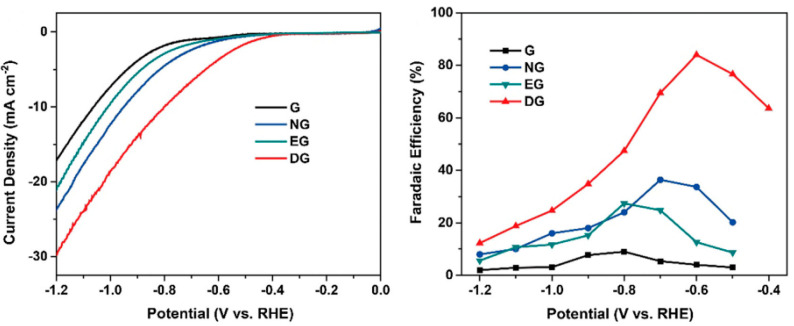
LSV curves in a 0.1 M KHCO_3_ electrolyte (the scan rate was 10 mVs^−1^) and faradaic efficiencies to CO at different applied potentials on pristine graphene, nitrogen-doped graphene (NG), edge-rich graphene (EG), and defective graphene (DG). Reproduced with permission [7].

Other significant work is reported by Han and col. that prepared graphene (G), nitrogen-doped graphene (NG), edge-rich graphene (EG), and defective graphene (DG). DG was synthesized through the elimination of the nitrogen atoms from an NG structure, which caused many topological defects that offered abundant catalytically active sites, high electronic conductivity, and strong CO_2_ adsorption [7]. This work revealed the great potential of DG as a metal-free GM for the ECO_2_RR. DG exhibited an excellent FE of 84% for CO production at −0.6 V (vs. RHE), with a smaller overpotential and a higher electrocatalytic activity toward the ECO_2_RR in comparison with the rest of materials (Figure 5).

**Figure 6:**
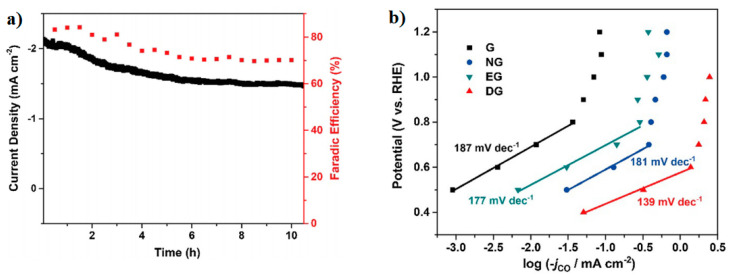
(**a**) Time-dependent total current density curve (left *y*-axis) and faradic efficiency for CO (right *y*-axis) at DG at −0.6 V (vs. RHE) in 0.1 M KHCO_3_. (**b**) Tafel plots for all catalysts. Reproduced with permission [7].

Furthermore, the DG electrocatalyst was relatively stable after 10 h of the continuous ECO_2_RR at −0.6 V (vs. RHE) (Figure 6a). In addition, Figure 6b reveals the operating mechanism and the best performance of the DG catalyst. Indeed, a Tafel slope value of 139 mV·dec^−1^ was obtained for DG, which indicates the initial electronic transfer to the adsorbed CO_2_ molecule to form the intermediate CO_2_^•−^ as the rate-determining step (RDS). The rest of the catalysts showed higher Tafel slope values that indicated the slower kinetics for the RDS [7].

### 2.2. Graphene-Supported Metal Nanoparticles

**Figure 7:**
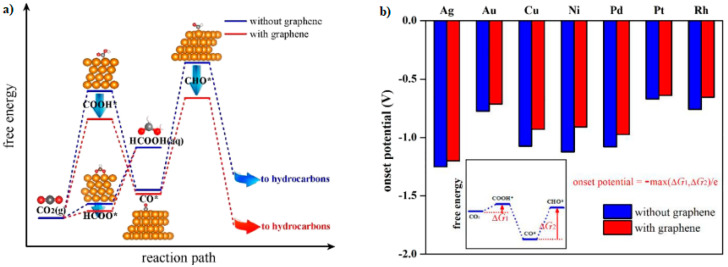
(**a**) A schematic free energy diagram. (**b**) Onset potentials for the electrochemical reduction reaction of carbon dioxide (ECO_2_RR) on metal surfaces with and without a graphene overlayer. Reproduced with permission [18].

The results described in the bibliography show that each metal catalyst exhibits differences in terms of efficiency and selectivity to reduce CO_2_ to different products. However, when graphene materials are used as metal support, there is a reduction in the free energy of the reaction in comparison with unsupported metal catalysts [16,18,27,37]. By applying DFT (density functional theory) calculations, Lin et al. showed that the interaction with graphene produces a strong influence on the formation of COOH* and CHO* species, as well as the suppression of HCOOH (Figure 7) [18]. With a higher Fermi level than CO_2_ molecules, the upper graphene layer injects electrons into the nonbonding orbitals of COOH* and CHO*, resulting in charge redistribution and electrostatic interaction between the molecules and graphene. The last reduces the free energies of the reaction and the onset potentials of crucial reaction steps (Figure 7b). Figure 7a shows the decrease in the free energy due to the incorporation of graphene; the reaction is consequently more thermodynamically favorable for both pathways (COOH* and CHO*), with the effect on the production of hydrocarbons being more significant in comparison with HCOOH [18]. Interestingly, these theoretical results have been confirmed with experimental studies [9,38].

**Figure 8:**
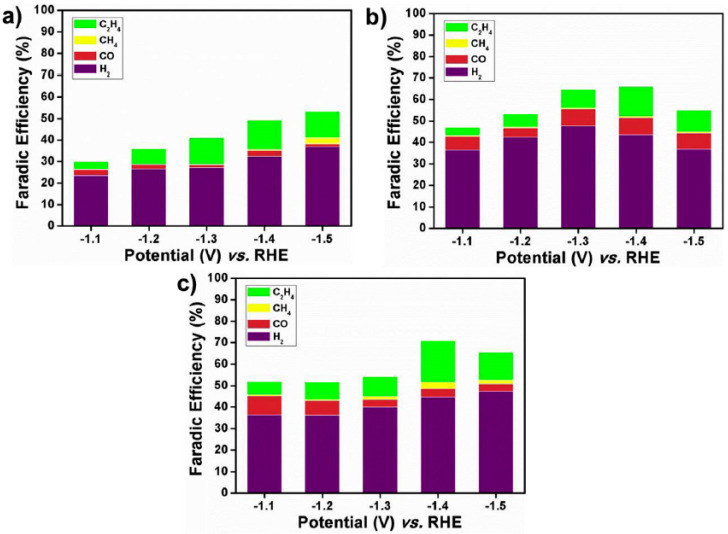
Faradaic efficiency of the gas products for (**a**) Cu_2_O, (**b**) Cu_2_O/rGO, and (**c**) Cu_2_O/N-rGO in a 0.1 M KHCO_3_ solution. Reproduced with permission [9].

Hossain et al. designed an easy procedure to synthesize a nanostructured thin film comprising NPs of Cu on rGO obtained by the direct electrochemical reduction of a mixture of copper and GO precursors [33]. The Cu (NP)/rGO film exhibited excellent stability and catalytic activity for the electrochemical reduction of CO_2_ in an aqueous solution, almost tripling the current densities in comparison with unsupported Cu NPs (Figure 3). Carbon monoxide and formate were found as the main products by chromatography, and the achieved FE was close to 69% at −0.6 V (vs. RHE, reference hydrogen electrode) [33]. On the other hand, nanocubes of cuprous oxide (Cu_2_O) incorporated into rGO and Cu_2_O (rGO) revealed promising results [9]. Figure 8a,b compare the FEs achieved at glassy carbon-supported Cu_2_O and Cu_2_O/rGO, respectively. The main outcomes indicated that the incorporation of the graphene material raised the production of CO and H_2_ and increased the stability of Cu_2_O nanocubes.

The incorporation of a second metal has been considered in theoretical calculations [31,39]. In order to modulate the interaction between the bimetallic catalyst and the graphene-based support (and therefore modify the catalytic activity), Cu, Ni, Pd, Pt, Ag, and Au were combined and compared with monometallic dimers by calculation [31]. It was found that Pt_2_, AgNi, Pd_2_, and AgPt supported on defective graphene revealed the lowest overpotential values for the ECO_2_RR [31]. On the other hand, transition metal dimers (Cu_2_, CuMn, and CuNi) introduced into single graphene vacancies (called @ 2SV) were studied for the ECO_2_RR by DFT [39]. All samples revealed a low overpotential, a high FE, and selectivity for species production during the ECO_2_RR. In this regard, Cu_2_ @ 2SV, MnCu @ 2SV, and NiCu @ 2SV promoted the formation of CO, CH_4_, and CH_3_OH, respectively. Results were explained in terms of the oxophilicity of Ni and Mn [39].

Bimetallic catalysts have also been employed in experimental studies. For instance, Pd-Cu/graphene catalysts with diverse Pd-Cu wt% ratios were synthesized from graphite oxide suspensions and metal precursor salts by applying the sodium borohydride reduction method [38]. Particle sizes ranging from 8 to 10 nm were acquired, and the best catalytic performance toward the ECO_2_RR was achieved with 1 wt% Pd–2 wt% Cu/graphene material [38].

In addition to copper-based catalysts, WC [13], Bi [17], In_2_O_3_ [37], and Pd-In [40] materials were supported on GMs, and a good catalytic performance toward the ECO_2_RR has been accomplished. For instance, three-dimensional Pd/graphene (Pd/3D-rGO), In/3D-rGO, and Pd-In/3D-rGO catalysts were prepared by a mild method that combined chemical and hydrothermal steps [40]. 3D-rGO materials are 3D structures with a high density of interconnected pores and metal NPs anchored on the folds. Interestingly, Pd_0.5_-In_0.5_/3D-rGO showed the smallest particle size, and its dispersion on rGO sheets was found to be more homogeneous than monometallic catalysts of Pd and In. These characteristics enhance the performance for the ECO_2_RR, decreasing the overpotential and increasing the FE to 85.3% at −1.6 V (vs. Ag/AgCl) in 0.5 M KHCO_3_ for formate production [40].

### 2.3. Metal Nanoparticles Supported on Heteroatom-Doped Graphene Materials

The introduction of heteroatoms into the graphene structure may improve the catalytic activity towards the ECO_2_RR. For example, Figure 8c shows that Cu_2_O supported on N-doped rGO (Cu_2_O/N-rGO) enhances the FE up to 70%, raises the production of ethylene at −1.4 V (vs. RHE), and increases the catalytic stability. Based on these results, the authors concluded that pyridine nitrogen improves the catalytic stability of Cu_2_O due to N (pyridine)–Cu interactions [9].

CuSn alloy nanoparticles supported on an NG material stand out due to their low cost and unique catalytic activities towards the ECO_2_RR. Nitrogen doping into graphene sheets results in the strong binding of CuSn NPs to the catalyst support. The strong binding prevents alloy NP detachment and agglomeration, thereby improving the long-term stability. Furthermore, electron transfer from NG to CuSn NPs appears to be beneficial for the adsorption of CO_2_ and/or hydrogen, thus enhancing the CO_2_RR performance [34].

**Figure 9:**
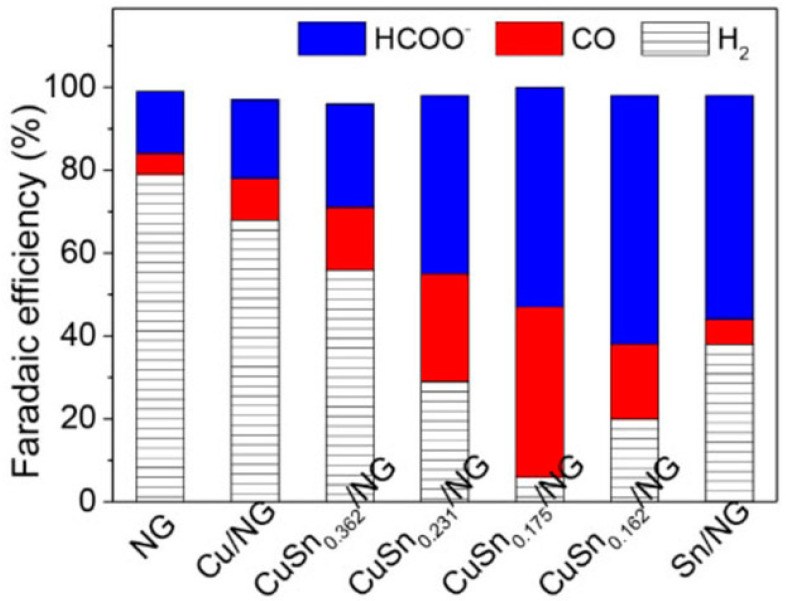
Faradaic efficiency of Cu-Sn NPs on NG with various ratios of Cu to Sn at −1.0 V (vs. RHE) for the ECO_2_RR (0.5 M KHCO_3_). Reproduced with permission [34].

**Figure 10:**
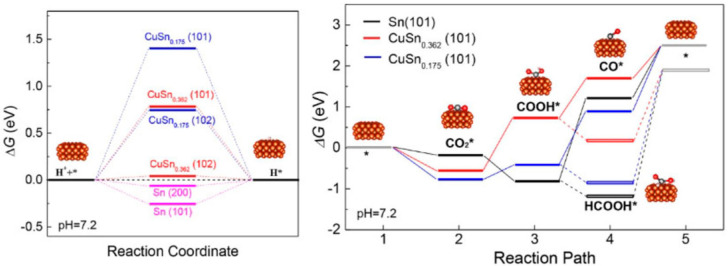
Left panel: Schematic Gibbs free energy profile for the H adsorption on different surfaces. Right panel: schematic Gibbs free energy profile for the detailed CO_2_RR pathway on the surface (101) of three models. Reproduced with permission [34].

Figure 9 depicts the distribution of products generated during this reaction as a function of the applied potential for different copper–tin ratios on NG, and an optimal composition was obtained for Cu-Sn_0.175_. This alloy generated C_1_ products with an FE close to 93% at −1.0 V (vs. RHE), which was the highest of all prepared materials. This led to considerable improvements over Cu and Sn separately, which were found to develop FEs of 32% and 58%, respectively [34]. To clarify the experimental results, DFT simulations of the free energy for hydrogen adsorption and CO_2_ reduction were carried out (Figure 10). The main results indicated that hydrogen can be easily adsorbed on Sn surfaces, but the free energies become higher after Cu introduction, and the HER is accordingly suppressed. After that, more H atoms can participate in the ECO_2_RR processes [34].

**Figure 11:**
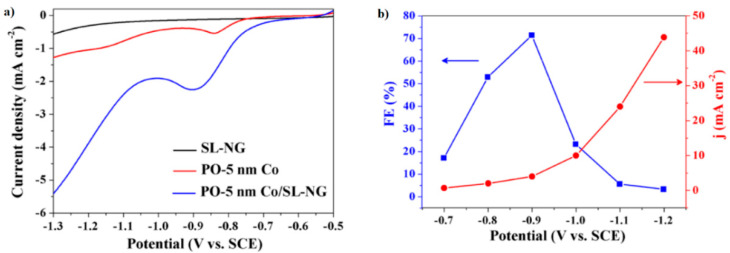
(**a**) Linear sweep voltammograms of single-layer nitrogen-doped graphene (SL-NG), 5-nm cobalt nanoparticles (PO-5 nm Co) and PO-5 nm Co/SL-NG in 0.1 M NaHCO_3_ at 20 mV s^−1^. (**b**) FE of methanol and current density for PO-5 nm Co/SL-NG under various electrolysis potentials for 10 h. Reproduced with permission [19].

On the other hand, Figure 11a illustrates the synergy between the catalyst and the catalyst support as an important increment of the faradaic current discerned at PO-5 nm Co/SL-NG catalyst. Additionally, the catalyst revealed remarkable stability, even after 10 h of CO_2_ electrolysis, both in current and in morphology, particle size, structure, and metal content, with an FE above 70% at −0.90 V (vs. SCE (saturated calomel electrode)) for methanol production (Figure 11b) [19].

**Figure 12:**
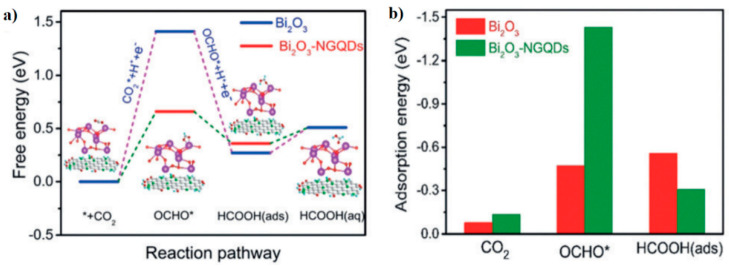
(**a**) Free-energy diagram and (**b**) adsorption energy of CO_2_, OCHO*, and HCOOH (ads) for Bi_2_O_3_ and Bi_2_O_3_-NGQDs. Reproduced with permission [35].

As stated above, Chen et al. developed a 2D/0D composite catalyst formed by Bi_2_O_3_-NGQDs [34]. This material exhibits an FE of almost 100% for formate production with a moderate potential of −1.0 V (vs. RHE) and good stability (Figure 4). Results were attributed through DFT calculations to an increment of the adsorption energy of CO_2_ and OCHO* after the combination of Bi_2_O_3_ with NGQDs (Figure 12b). Figure 12a illustrates the free energy diagram for the HCOOH formation process on Bi_2_O_3_ and Bi_2_O_3_-NGQDs materials, which reveals the highest energy barrier for the OCHO* formation that intensely falls for Bi_2_O_3_-NGQDs. Furthermore, the energy barrier for the HER increases for MG, thus explaining the preference of Bi_2_O_3_-NGQDs material to generate formate over H_2_ [35].

## 3. Perspective

In this review, the general concept of the CO_2_ reduction reaction was discussed, with special attention paid to low temperature electrolysis with graphene-based electrode materials.

The conversion of CO_2_ to chemicals usable as fuels via room temperature electrolysis with graphene-based catalysts is a very attractive technology for energy conversion and storage. This technology has the potential to reduce the produced CO_2_ through its conversion into valuable energy carriers (hydrocarbons, alcohols, or CO-rich feeds) that will help to improve environmental contamination.

With the aim to solve the principal catalytic problem at the cathodes of low temperature electrolyzers, the fundamental study of carbon dioxide, as well as hydrogen evolution on graphene-based materials in a wide pH range, was considered.

In general, the results discussed in this manuscript confirm that graphene materials are excellent supports, producing synergistic effects that cause improvements in the efficiency and selectivity of the catalysts used for the electrochemical reduction of CO_2_. However, in most cases, the activity of graphene not modified by metal species is very low. All the new advances in the fundamental understanding of reactions occurring on graphene-based catalysts presented here may help to improve the fabrication of novel electrodes in order to enhance the performance and decrease the cost of this technology.
